# Sphingomyelin Biosynthesis Is Essential for Phagocytic Signaling during Mycobacterium tuberculosis Host Cell Entry

**DOI:** 10.1128/mBio.03141-20

**Published:** 2021-01-26

**Authors:** Patrick Niekamp, Gaelen Guzman, Hans C. Leier, Ali Rashidfarrokhi, Veronica Richina, Fabian Pott, Caroline Barisch, Joost C. M. Holthuis, Fikadu G. Tafesse

**Affiliations:** a Department of Molecular Microbiology and Immunology, Oregon Health & Science University, Portland, Oregon, USA; b Molecular Infection Biology Division, Department of Biology and Chemistry, University of Osnabrück, Osnabrück, Germany; c Molecular Cell Biology Division, Department of Biology and Chemistry, University of Osnabrück, Osnabrück, Germany; Washington University School of Medicine in St. Louis

**Keywords:** cell signaling, *Mycobacterium tuberculosis*, sphingolipids, sphingomyelin, phagocytosis

## Abstract

Phagocytosis by alveolar macrophages is the obligate first step in Mycobacterium tuberculosis (*Mtb*) infection, yet the mechanism underlying this process is incompletely understood. Here, we show that *Mtb* invasion relies on an intact sphingolipid biosynthetic pathway. Inhibition or knockout of early sphingolipid biosynthetic enzymes greatly reduces *Mtb* uptake across multiple phagocytic cell types without affecting other forms of endocytosis. While the phagocytic receptor dectin-1 undergoes normal clustering at the pathogen contact sites, sphingolipid biosynthetic mutant cells fail to segregate the regulatory phosphatase CD45 from the clustered receptors. Blocking sphingolipid production also impairs downstream activation of Rho GTPases, actin dynamics, and phosphoinositide turnover at the nascent phagocytic cup. Moreover, we found that production of sphingomyelin, not glycosphingolipids, is essential for *Mtb* uptake. Collectively, our data support a critical role of sphingomyelin biosynthesis in an early stage of *Mtb* infection and provide novel insights into the mechanism underlying phagocytic entry of this pathogen.

## Abstract (precis)

Mycobacterium tuberculosis (*Mtb*) invades alveolar macrophages through phagocytosis to establish infection and cause disease. The molecular mechanisms underlying *Mtb* entry are still poorly understood.

## INTRODUCTION

Mycobacterium tuberculosis (*Mtb*), the etiologic agent of tuberculosis (TB), remains a significant global health threat. It is estimated that more than two billion people around the world are infected with the latent form of *Mtb* and about 1.5 million die every year (1). The success of *Mtb* as a pathogen relies on its ability to effectively enter the immune cells and establish its niche by subverting the defense mechanisms of the host (2, 3). Phagocytosis of *Mtb* by lung-resident alveolar macrophages represents the obligate first step in the *Mtb* infection cycle. *Mtb* can proliferate in these cells, escape from the phagosome, and migrate to local lymph nodes to disseminate and spread the infection (4). Precise knowledge of the mechanism exploited by *Mtb* to invade immune cells is desirable, as this may yield novel targets and treatment options to combat TB.

Macrophages, along with dendritic cells and neutrophils, serve as professional phagocytic cells of the immune system that are specialized in the recognition, engulfment, and elimination of microbial pathogens, thereby contributing to the first line of defense against infection. Phagocytosis is a receptor-initiated process involving a massive reorganization and deformation of the cell membrane coordinated by the underlying actin cytoskeleton to enable the internalization of microbial pathogens (5). To this end, phagocytes are equipped with pattern recognition receptors (PRRs) that bind ligands on the surface of the pathogen (6, 7). Prototypical PRRs include Toll-like receptors, scavenger receptors and C-type lectin receptors of the dectin family. Activation of PRRs results from the clustering of several receptors upon binding to multiple adjacent ligands on the pathogen surface, which brings their cytosolic domains in close apposition. This initiates signaling by nonreceptor tyrosine kinases of the Src family and the recruitment of phosphoinositide-modifying enzymes (6, 8). Local alterations in phosphoinositide levels and concomitant activation of Rho family GTPases orchestrate a remodeling of the actin cytoskeleton, which drives an extension of the plasma membrane along the particle to form a phagocytic cup. Further actin polymerization and contractile activity lead to membrane apposition and eventual sealing of the nascent phagosome, which then evolves into a degradative phagolysosome (6, 8). While the precise mechanism by which *Mtb* invades immune cells remains to be established, a wide variety of PPRs has been implicated in the recognition and phagocytic uptake of mycobacteria (9–11). This apparent redundancy in the entry process hampers the development of an effective strategy to target a critical step in the infection cycle of *Mtb*.

Sphingolipids are essential lipids enriched in the plasma membrane of mammalian cells, where they interact with cholesterol to control vital membrane properties, serve as adhesion sites for extracellular proteins, and play important roles in signal transmission and cell recognition (12–14). Sphingolipids have also emerged as active participants in various infectious diseases. Various pathogens, including bacteria, fungi, and viruses, modulate host membrane sphingolipids and their metabolites to manipulate host defense, thereby promoting their survival and pathogenicity (15). For instance, Neisseria gonorrhoeae and Pseudomonas aeruginosa exploit the hydrolysis of cell surface sphingomyelin (SM) to generate ceramide-rich microdomains that serve as portals for their entry into macrophages (16, 17). Sphingolipids also play a critical role in the *in vivo* clearance of the pathogenic fungi Candida albicans through phagocytosis (18). Moreover, mycobacteria utilize host sphingolipids and cholesterol as nutrient sources for their growth and survival (19). Although the role of host membrane cholesterol in the entry of different *Mycobacterium* species was addressed previously (20), the function of sphingolipids in *Mtb* invasion has not been explored.

Here, we show that blocking sphingolipid biosynthesis causes a substantial reduction in the uptake of both *Mtb* and Mycobacterium marinum by a panel of macrophages and dendritic cells without affecting clathrin-mediated endocytosis or micropinocytosis. Time-lapse microscopy revealed that sphingolipid-deficient macrophages are capable of pathogen recognition and actin assembly at the contact site but impaired in the subsequent remodeling of the actin cytoskeleton necessary to complete internalization of the pathogen. We also show that sphingolipid-deficient macrophages fail to exclude the regulatory tyrosine phosphatase CD45 from the receptor-pathogen contact site, a crucial step in the development of a functional phagocytic synapse. This defect was accompanied by an impaired activation of Rho GTPases and significant reduction in phosphoinositide turnover. Finally, we show that the production of SM, not glycosphingolipids, is essential for efficient *Mtb* uptake. Our findings offer fresh mechanistic insights into how *Mtb* invades immune cells and establish a critical role for SM in this process.

## RESULTS

### Manipulation of sphingolipid levels in mammalian phagocytes.

**FIG 1:**
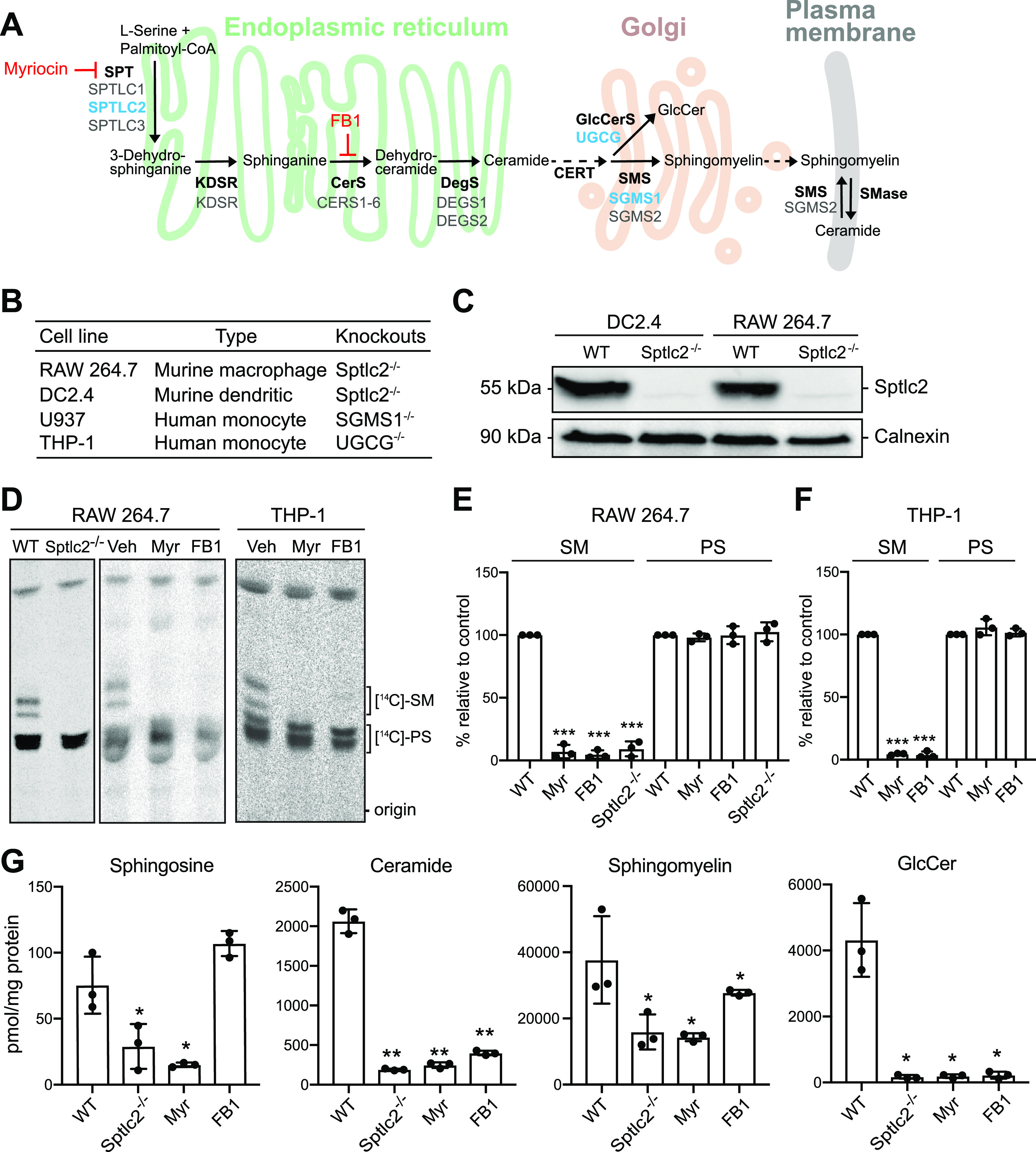
Manipulation of cellular sphingolipid levels using genetics and chemical tools. (A) Schematic outline of the sphingolipid biosynthetic pathway. Inhibitors of sphingolipid biosynthetic enzymes used in this study are in red. Genes deleted using CRISPR/Cas9 are in blue. SPT, serine palmitoyltransferase; KDSR, 3-ketodihydrosphingosine reductase; CerS, ceramide synthase; DegS, δ4-desaturase; GlcCerS, glucosylceramide synthase; SMS, sphingomyelin synthase; FB1, fumonisin B1. (B) Phagocytic cell lines used in this study. (C) DC2.4 and RAW264.7 wild-type (WT) and Sptlc2^−/−^ cells were processed for immunoblotting with antibodies against Sptlc2 and calnexin. (D) Sptlc2^−/−^ and wild-type RAW264.7 or THP-1 cells were treated with myriocin or fumonisin B1 for 3 days and then metabolically labeled with 3-l-[^14^C]serine for 4 h. Total lipids were extracted and analyzed by thin-layer chromatography (TLC) and autoradiography. Veh, vehicle treated. (E and F) Quantification of [^14^C]SM and [^14^C]PS signals from TLCs shown in panel D. (G) Wild-type, Sptlc2^−/−^, and inhibitor-treated RAW264.7 cells were subjected to total lipid extraction. Sphingosine, ceramide, sphingomyelin, and glucosylceramide (GlucCer) levels were quantified by LC-MS/MS. Data are means ± standard deviations (SD) (*n *= 3). *, *P* < 0.05; **, *P* < 0.01; ***, *P* < 0.001 (two-tailed unpaired *t* test).

As depicted in Fig. 1A, the initial and rate-limiting step of sphingolipid biosynthesis is the production of a sphingoid base through the condensation of serine and palmitoyl coenzyme A (palmitoyl-CoA), a reaction performed by the serine palmitoyltransferase (SPT) enzyme complex (21). Genetic ablation of the SPT subunit Sptlc2 blocks the *de novo* synthesis of all sphingolipids, as does treatment with the atypical amino acid myriocin (Myr), a potent inhibitor of SPT activity (18). The fungal toxin fumonisin B1 (FB1) acts several steps downstream in the sphingolipid biosynthetic pathway by blocking the activity of ceramide synthases (CerS) (18, 22) As the central nexus of sphingolipid biosynthesis, ceramide is converted into a variety of complex sphingolipids, including sphingomyelin (SM) and glycosphingolipids (23, 24). To define the role of sphingolipid biosynthesis in the phagocytic uptake of *Mtb*, we employed four different phagocytic cell lines: murine RAW246.7 macrophages, murine DC2.4 dendritic cells, and human THP-1 and U937 monocytes (Fig. 1B).

To verify the reported effects of Myr and FB1 on *de novo* sphingolipid biosynthesis, we monitored the incorporation of 3-l-[^14^C]serine into sphingolipid species in RAW246.7, THP-1, DC2.4, and U937 cells after 3 days of drug treatment using thin-layer chromatography (TLC) analysis. Across all four cell lines examined, treatment with Myr or FB1 in each case reduced incorporation of the serine isotope into SM by more than 90%; in contrast, incorporation of the isotope into phosphatidylserine (PS) was largely unaffected (Fig. 1D to F; also, see Fig. S1A and B in the supplemental material). Genetic ablation of Sptlc2 in RAW246.7 and DC2.4 cells also virtually abolished *de novo* SM production without affecting PS biosynthesis (Fig. 1C to E; Fig. S1A), in line with our previous findings (18).

10.1128/mBio.03141-20.1 FIG S1 Blocking *de novo* sphingolipid biosynthesis using genetics and chemical tools has no significant impact on cell growth. Sptlc2^−/−^ and wild-type DC2.4 or U937 cells were treated with myriocin or fumonisin B1 for 3 days and then metabolically labelled with 3-l-[^14^C]serine for 4 h. Total lipids were extracted and analyzed by TLC and autoradiography. Data are representative of 3 independent experiments. Veh, vehicle-treated. (C) Phase-contrast microscopy images of wild-type and Sptlc2^−/−^ RAW264.7 cells. (D) Numbers of wild-type and Sptlc2^−/−^ RAW264.7 cells plated out at equal densities and grown for 24 or 48 h in serum-containing medium. Data are means ± SD (*n *= 3). Download FIG S1, TIF file, 2.0 MB. Copyright © 2021 Niekamp et al. 2021 Niekamp et al. This content is distributed under the terms of the Creative Commons Attribution 4.0 International license.

We next examined the impact of drug treatment and Sptlc2 removal on the steady-state sphingolipid levels. Both Myr-treated and Sptlc2^−/−^ cells contained significantly (≥3-fold) reduced levels of sphingosine, ceramides, SM, and glucosylceramide (GlcCer) in comparison to control cells (Fig. 1G). In FB1-treated cells, we observed a reduction in the levels of ceramide, SM, and GlcCer concomitant with an increase in sphingosine levels, as expected (Fig. 1G) (25). The residual sphingolipid levels detected in Sptlc2^−/−^, Myr-treated, and FB1-treated cells are likely due to the activity of sphingolipid salvage pathways, through which cells may acquire sphingolipids from additives in the culture medium—primarily serum. This enables them to bypass the growth defect that is generally observed when sphingolipid biosynthetic mutants are cultured in serum-depleted medium. Indeed, we observed that RAW264.7 and DC2.4 cells lacking Sptlc2 grew equally well in serum-containing medium as their wild-type counterparts (Fig. S1C and D) (18).

### Sphingolipid-deficient cells display a reduced capacity to phagocytose *Mtb*.

**FIG 2:**
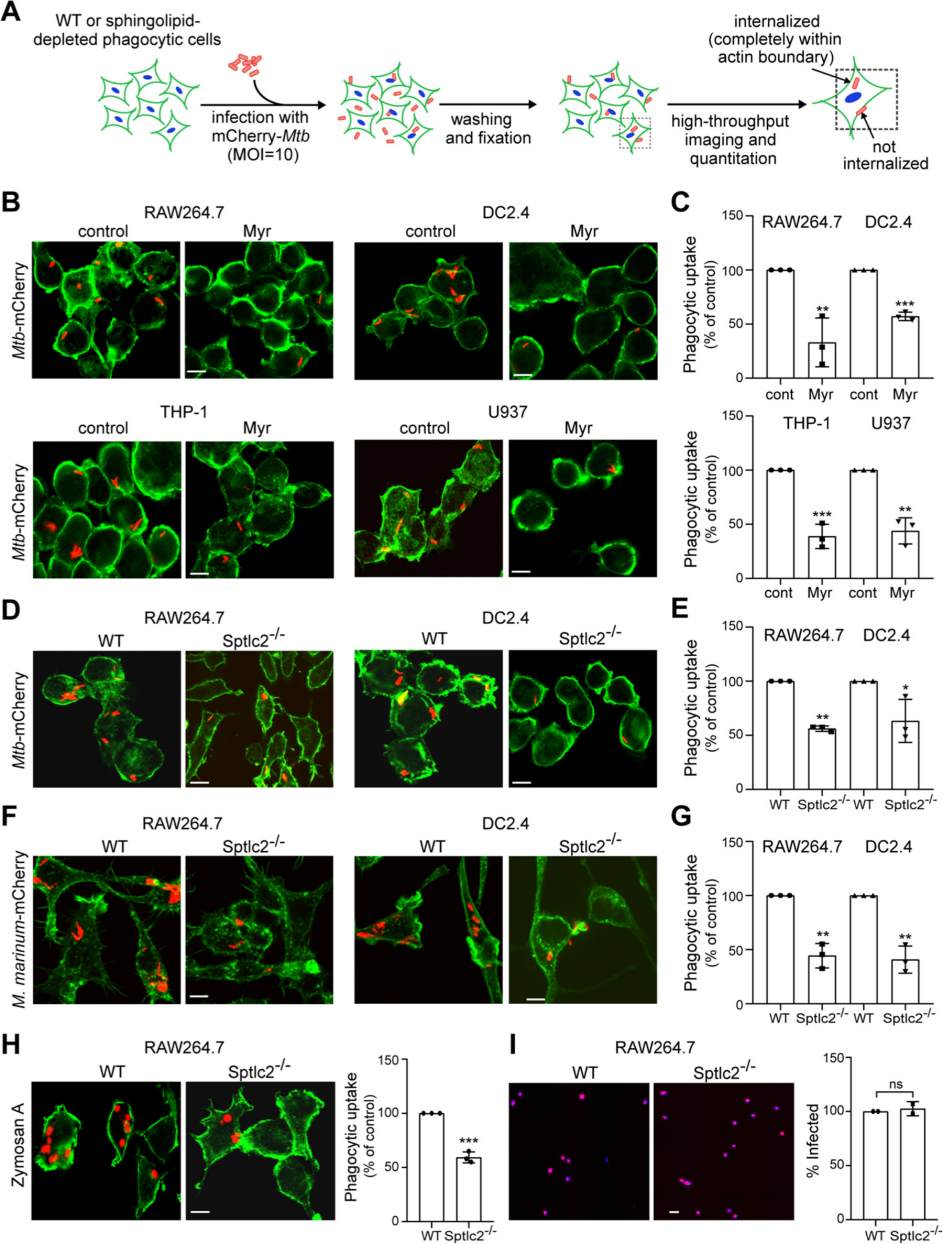
Sphingolipid biosynthesis is required for efficient phagocytosis of *Mtb*. (A) Control and sphingolipid-depleted phagocytic cells were infected with mCherry-expressing *Mtb* at an MOI of 10 for 2 h, washed, fixed, stained with phalloidin Alexa Fluor 488 and DAPI, and then analyzed by high-throughput fluorescence imaging. The efficiency of phagocytic uptake was determined by dividing the total number of internalized bacteria by the total number of cells. (B) RAW264.7, DC2.4, THP-1, and U937 cells were grown in the presence or absence of myriocin for 3 days, infected with mCherry-expressing *Mtb*, and then processed as for panel A. (C) Quantification of *Mtb* uptake by cells treated as for panel B. (D) Wild-type and Sptlc2^−/−^ RAW264.7 and DC2.4 cells were infected with mCherry-expressing *Mtb* and then processed as for panel A. (E) Quantification of *Mtb* uptake by cells treated as for panel D. (F) Wild-type and Sptlc2^−/−^ RAW264.7 and DC2.4 cells were infected with mCherry-expressing M. marinum at an MOI of 10 for 2 h and then processed as for panel A. (G) Quantification of M. marinum uptake by cells treated as for panel F. (H) Wild-type and Sptlc2^−/−^ RAW264.7 cells were incubated with zymosan A particles at an MOI of 1 for 1 h and then processed as for panel A. (I) Representative images of wild-type and Sptlc2^−/−^ RAW264.7 cells infected with mCherry-tagged herpes simplex virus (HSV) at an MOI of 1. At 12 h postinfection, cells were fixed, stained with DAPI, and analyzed by high-throughput fluorescence microscopy to determine the percent infected cells. Bar, 5 μm. Data in panels C, E, and G are means ± SD (*n *= 3). *, *P* < 0.05; **, *P* < 0.01, ***, *P* < 0.001 (unpaired *t* test). Data in panel I are means ± standard errors (SE) (*n *= 2). ns, not significant by two-tailed unpaired *t* test.

To address whether sphingolipids participate in the phagocytic uptake of *Mtb*, RAW264.7, THP-1, DC2.4, and U937 cells were treated with Myr and FB1 for 3 days and then infected with an mCherry-expressing strain of *Mtb*. In brief, cells were infected at a multiplicity of infection (MOI) of 10 for 2 h, fixed, and then stained with DyLight 488-conjugated phalloidin and DAPI (4′,6-diamidino-2-phenylindole) to visualize filamentous actin (F-actin) and cell nuclei, respectively. Fluorescence microscopy was used to quantify uptake efficiency by automated counting of cell number (blue nuclei) and internalized bacteria (red fluorescent particles within green cell boundary) (Fig. 2A). Uptake efficiency was defined as the ratio of the total number of internalized *Mtb* particles to the total number of identified nuclei. The ratio is reported as a percentage of phagocytosis relative to that of the control cells. In all four cell models analyzed, we observed that Myr and FB1 treatment caused a significant (40 to 60%) reduction in *Mtb* uptake (Fig. 2B and C; Fig. S2A to D). Similarly, genetic ablation of Sptlc2 in RAW264.7 and DC2.4 cells results in an ∼50% reduction in uptake relative to their wild-type controls (Fig. 2D and E). Collectively, these results indicate that an intact sphingolipid biosynthetic pathway is essential for an efficient uptake of *Mtb* by professional phagocytes. To test whether the same holds true for other mycobacteria, we used Mycobacterium marinum, a natural pathogen of poikilotherms that is closely related to *Mtb* and serves as a widely accepted model for TB (26, 27). Similar to infections with *Mtb*, Sptlc2^−/−^ RAW264.7 and DC2.4 cells showed an ∼50% reduction in phagocytic uptake of M. marinum relative to wild-type cells (Fig. 2F and G).

10.1128/mBio.03141-20.2 FIG S2 Sphingolipid biosynthesis is required for efficient phagocytosis of *Mtb* but not for receptor-mediated uptake of transferrin. (A and C) RAW264.7, DC2.4, THP-1, and U937 cells were grown in the presence or absence of fumonisin B1 for 3 days, infected with mCherry-expressing *Mtb* for 2 h, washed, fixed, stained with phalloidin Alexa Fluor 488 and DAPI, and then imaged by fluorescence microscopy. (B and D) Quantification of *Mtb* uptake by cells treated as for panel A. (E and F) Sptlc2^−/−^ cells bind but fail to internalize pathogen-mimicking beads; (E) Wild-type and Sptlc2^−/−^ RAW264.7 cells were incubated with rabbit IgG-opsonized zymosan A particles for 5 min at 37°C. Next, cells were washed with chilled PBS and stained at 4°C with donkey anti-rabbit IgG-Cy3 in PBS supplemented with 1% BSA for 15 min to distinguish external beads (red) from internalized beads (blue). After chemical fixation, cells were stained for actin with phalloidin Alexa Fluor 488 (green) and analyzed by confocal fluorescence microscopy. (F) Quantification of the ratio of internalized (blue) over attached beads (red). Three independent experiments were performed, each in triplicate, with at least 100 cells quantified per replicate. (G) Differentiated THP-1 cells were incubated with 100 nM Alexa Fluor 555-conjugated human transferrin (Tf555) in the absence or presence of different concentrations of nonfluorescent transferrin for 30 min. Next, cells were washed in acidic buffer and fixed, and receptor-mediated uptake of Tf555 was quantified using a plate reader fluorimeter. (H) Quantification of receptor-mediated uptake of Tf555 in control or 3-day myriocin-treated THP-1 cells. Data are means ± SD (*n *= 3). **, *P* < 0.01; *, *P* < 0.05; ns, not significant (unpaired *t* test). Download FIG S2, TIF file, 0.4 MB. Copyright © 2021 Niekamp et al. 2021 Niekamp et al. This content is distributed under the terms of the Creative Commons Attribution 4.0 International license.

### Sptlc2^−/−^ cells are permissive to herpes simplex virus entry and clathrin-mediated endocytosis.

To determine whether an intact sphingolipid biosynthetic pathway is a general prerequisite for the entry of pathogens in professional phagocytes, we performed parallel infections of wild-type and Sptlc2^−/−^ RAW264.7 cells with a fluorescent reporter strain of herpes simplex virus 1 (HSV-1) encoding a fusion of the viral protein VP26 and mCherry (28). HSV-1 is an enveloped virus that gains entry to host cells through the phagocytosis-like process of micropinocytosis; the viral receptor involved is nectin-1, also known as herpesviruses entry mediator (29, 30). At 12 h postinfection, we quantified the rates of HSV-1 infection using high-content imaging as described above. Because the VP26-mCherry reporter construct localizes to the nucleus, we did not stain cells using phalloidin and instead used the colocalization of DAPI with mCherry to determine infection efficiency. However, we observed no significant difference in the HSV-1 infection rates between wild-type and Sptlc2^−/−^ cells (Fig. 2I). We also analyzed Myr-treated and control THP-1 cells for their ability to take up transferrin (Tf), which binds Tf receptors on the cell surface to enter cells via clathrin-mediated endocytosis (31). As shown in Fig. S2G and H, Myr treatment did not affect the endocytic uptake of transferrin. Collectively, these data indicate that sphingolipid biosynthesis is dispensable for HSV-1 entry and clathrin-mediated endocytosis, thus supporting a more specific role of sphingolipids in the phagocytic uptake of *Mtb*.

### Sptlc2^−/−^ cells display defects in filamentous actin dynamics during phagocytic uptake.

**FIG 3:**
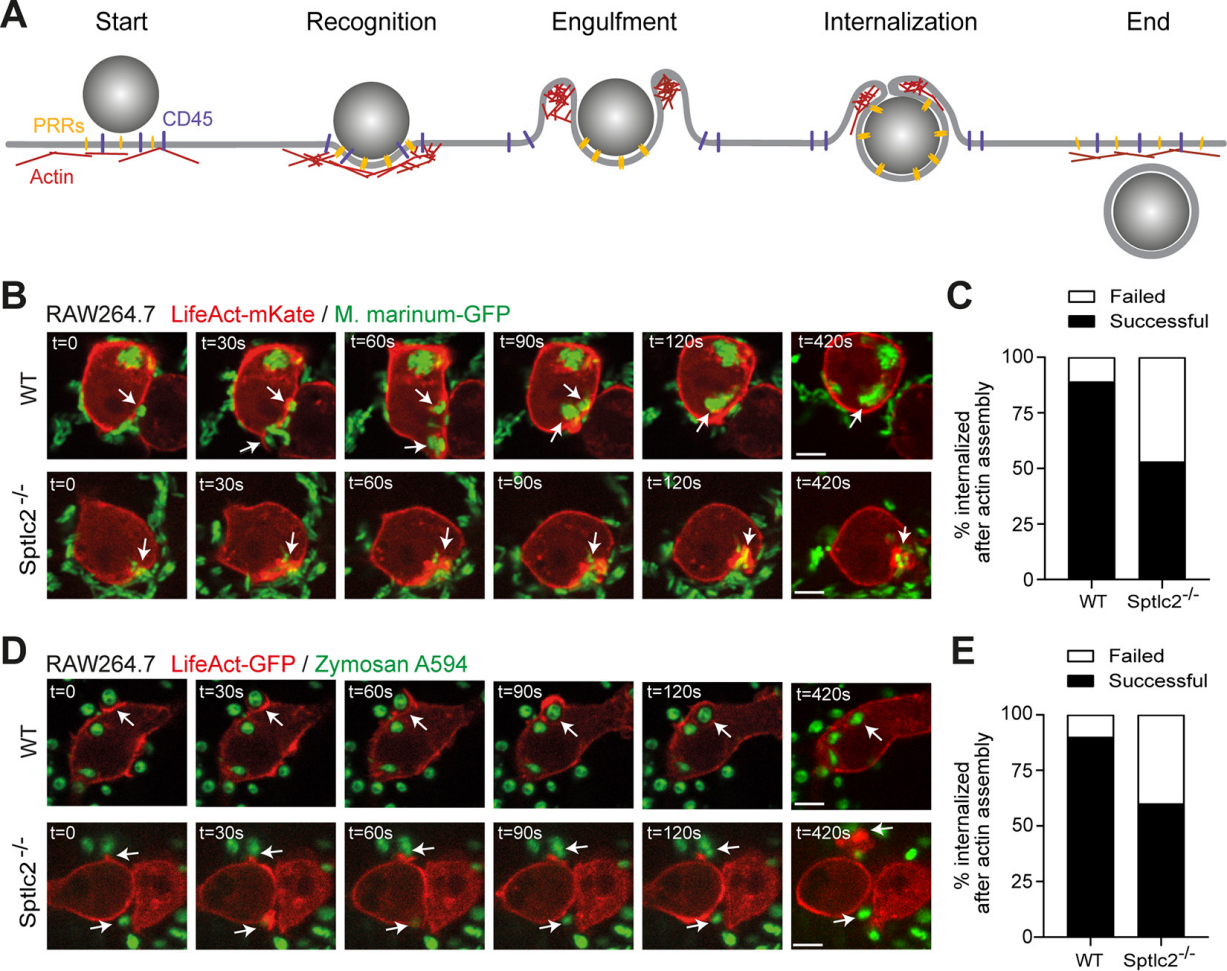
Sptlc2^−/−^ cells display a defect in actin dynamics during phagocytic cup formation. (A) Schematic outline of F-actin remodeling during phagocytosis. Clustering of pattern recognition receptors (PRRs) at the pathogen interaction site generates second messengers that promote local actin polymerization. Actin continues to be actively polymerized to extend the phagosomal membrane while being remodeled at the base of the phagocytic cup. Further actin polymerization and contractile activity lead to membrane apposition and eventually fusion to complete phagosome formation. (B) Time-lapse confocal images of wild-type and Sptlc2^−/−^ RAW264.7 cells stably transduced with LifeAct-mKate (red) and infected with GFP-expressing M. marinum (green). Arrows indicate sites where mycobacteria trigger actin assembly, initiating formation of a phagocytic cup. Unlike wild-type cells, Sptlc2^−/−^ RAW264.7 cells often failed to disassemble actin at the base of phagocytic cups, thus blocking entry of the mycobacteria. (C) Quantification of the percentage of successful versus failed phagocytic uptake events of M. marinum in wild-type and Sptlc2^−/−^ RAW264.7 cells. (D) Time-lapse confocal images of wild-type and Sptlc2^−/−^ RAW264.7 cells stably transduced with LifeAct-GFP (red) and incubated with zymosan A particles (green). Bar, 5 μm. (E) Quantification of the percentage of successful versus failed phagocytic uptake events of zymosan A particles in wild-type and Sptlc2^−/−^ RAW264.7 cells. Data represent uptake events in >30 cells per condition. Bar, 5 μm.

Particle ingestion by phagocytosis results from sequential rearrangements of F-actin and the overlying membrane. F-actin polymerization is the force driving membrane extension and phagosome formation. Nucleation of filaments at the site of particle recognition initiates F-actin polymerization and promotes formation of pseudopodia or lamellae. Then, actin filaments depolymerize at the base of the phagocytic cup, while polymerization proceeds in the tips of the lamellae to promote phagosomal membrane extension and closure (32) (Fig. 3A). To determine at which step in this process sphingolipids exert their critical role, we monitored F-actin dynamics in wild-type and Sptlc2^−/−^ RAW264.7 cells expressing LifeAct-mKate during infection with a monomeric GFP (mGFP)-expressing strain of M. marinum. Time-lapse fluorescence microscopy revealed that in wild-type RAW264.7 cells, F-actin readily assembled at sites of pathogen binding (Fig. 3B; Movie S1). In nearly all cases (∼89%; *n *= 46), this led to a successful internalization of the pathogen and clearance of F-actin polymers (Fig. 3C). In contrast, while RAW264.7 Sptlc2^−/−^ cells readily assembled F-actin at the site of pathogen recognition, in about half of the cases (47%; *n *= 30) the pathogen failed to enter and F-actin remained assembled at the contact site (Fig. 3B and C; Movie S1).

10.1128/mBio.03141-20.4 MOVIE S1 Time-lapse confocal images of a wild-type (left) and Sptlc2^−/−^ (right) RAW264.7 cell stably transduced with LifeAct-mKate (red) and infected with GFP-expressing M. marinum (green). Images were taken every 15 s. Bar, 5 μm. Download Movie S1, AVI file, 0.2 MB. Copyright © 2021 Niekamp et al. 2021 Niekamp et al. This content is distributed under the terms of the Creative Commons Attribution 4.0 International license.

An in-depth microscopic analysis of the role of sphingolipids in the phagocytic uptake of *Mtb* and M. marinum is hampered by the fact that these pathogens are often internalized as heterologous clusters that comprise multiple bacteria. To overcome this limitation, we also analyzed wild-type and Sptlc2^−/−^ RAW264.7 cells for their ability to take up zymosan A beads. These pathogen-mimicking particles engage the C-type lectin receptor dectin-1 (18, 33, 34), which is one of several pathogen recognition receptors on professional phagocytes that recognize *Mtb* and initiate phagocytic signaling to facilitate host cell entry (35, 36). Sptlc2^−/−^ RAW264.7 cells displayed an ∼40% reduction in the phagocytic uptake of zymosan A particles relative to wild-type cells (Fig. 2H), similar to the defect in *Mtb* and M. marinum uptake observed in these cells (Fig. 2D to G). Time-lapse microscopy revealed that in wild-type RAW264.7 cells, zymosan A particles initiating F-actin assembly at the recognition site were efficiently internalized, whereby depolymerization of F-actin at the base of the phagocytic cup and subsequent F-actin polymerization in the leading edges of the lamellae could be readily visualized in real time (90%; *n *= 30) (Fig. 3D and E; Movie S2). Again, while RAW264.7 Sptlc2^−/−^ cells readily assembled F-actin at the site of particle recognition, in a significant number of cases the particles failed to enter (40%; *n *= 30) and F-actin polymers remained assembled at the contact site (Fig. 3D and E; Movie S2). In a complementary approach, wild-type and Sptlc2^−/−^ RAW264.7 cells were incubated with IgG-opsonized zymosan A particles for 10 min and cell surface-bound particles were discriminated from internalized particles by immunostaining. Sites of F-actin polymerization were visualized by labeling cells with phalloidin Alexa Fluor 488. As shown in Fig. S2E and F, Sptlc2^−/−^ cells displayed a nearly 3-fold reduction in the ratio of internalized to cell surface-bound beads compared to wild-type cells. Together, these results indicate that sphingolipids are dispensable for the initial steps of phagocytosis, including pathogen recognition and the assembly of F-actin polymers at the contact site. Instead, it appears sphingolipids exert their critical role immediately downstream of these events, as Sptlc2^−/−^ cells often fail to complete the sequential rearrangements of F-actin necessary for pathogen entry.

10.1128/mBio.03141-20.5 MOVIE S2 Time-lapse confocal images of a wild-type (left) and Sptlc2^−/−^ (right) RAW264.7 cell stably transduced with LifeAct-mKate (red) and incubated with zymosan A particles (green). Images were taken every 15 s. Bar, 5 μm. Download Movie S2, AVI file, 0.2 MB. Copyright © 2021 Niekamp et al. 2021 Niekamp et al. This content is distributed under the terms of the Creative Commons Attribution 4.0 International license.

### Sptlc2^−/−^ cells fail to exclude CD45 from the pathogen contact site.

**FIG 4:**
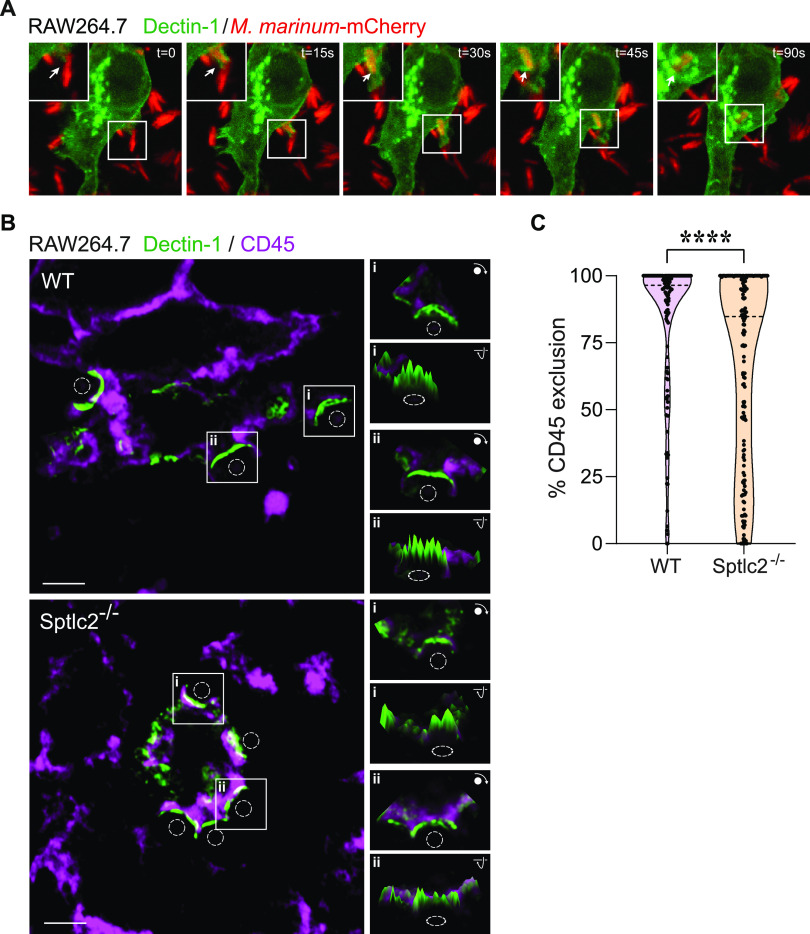
Segregation of CD45 from dectin-1 at the phagocytic cup is affected in Sptlc2^−/−^ cells. (A) Time-lapse confocal images of wild-type RAW264.7 cells transfected with GFP-tagged dectin-1 (green) and infected with mCherry-expressing M. marinum (red). Arrows indicate sites where mycobacteria trigger clustering of dectin-1. Bar, 5 μm. (B) Wild-type and Sptlc2^−/−^ RAW264.7 cells transfected with GFP-tagged dectin-1 (green) were incubated with zymosan A particles (dashed circles) on ice, warmed to 37°C for 2 min to initiate phagocytosis, fixed, and then stained with antibodies against CD45 (purple). Representative slices of z-stacks are shown. Insets show fluorescence intensity profiles of CD45 and dectin-1-GFP signals in a single slice. Bar, 3 μm. (C) Violin plots showing the level of CD45 segregation from dectin-1-enriched phagocytic cups in wild-type and Sptlc2^−/−^ RAW264.7 cells, defined as the percentage of dectin-1 volume containing no CD45 voxels. More than 100 individual cups were analyzed per cell line. ****, *P = *0.00006 by the two-tailed unpaired *t* test.

The engagement of PPRs with their ligands initiates receptor clustering at the phagocytic cup, followed by a lateral segregation of the inhibitory phosphatase CD45 (8). Because sphingolipids have been implicated as active participants in the organization of signaling complexes at the cell surface (37), we reasoned that sphingolipid levels at the plasma membrane may be critical for the aggregation of dectin-1 and/or exclusion of CD45 from that pathogen contact site. To experimentally address this idea, we transfected wild-type and Sptlc2^−/−^ RAW264.7 cells with GFP-tagged dectin-1 and assessed the colocalization of the fluorescent receptor with CD45 following treatment with nonfluorescent zymosan A beads. As control, we also monitored the subcellular distribution of GFP-tagged dectin-1 in RAW264.7 cells upon infection with mCherry-expressing M. marinum. Dectin-1 readily accumulated at the particle contact site in M. marinum*-*exposed cells (Fig. 4A; Fig. S3A; Movies S3 and S4). Besides supporting a role of dectin-1 in the phagocytic uptake of mycobacteria, these findings point to a significant overlap in the pathways that mediate host cell entry of zymosan A beads and mycobacteria. In both wild-type and Sptlc2^−/−^ cells, dectin-1 aggregated at the zymosan A contact site within 2 min after bead exposure, indicating that sphingolipid biosynthesis is dispensable for ligand-induced receptor clustering. As expected, in wild-type cells, the inhibitory phosphatase CD45 was efficiently segregated from the dectin-1 clusters at the particle contact site (Fig. 4B and C; Fig. S3B) (8). In contrast, CD45 failed to efficiently segregate from the dectin-1 clusters in zymosan A-treated Sptlc2^−/−^ cells (Fig. 4B and C).

10.1128/mBio.03141-20.3 FIG S3 (A) WT RAW264.7 cells transfected with GFP-tagged dectin-1 (green) were infected with mCherry-expressing M. marinum (red) as for Fig. 4A. Bar, 5 μm. Data are means ± SD (*n *= 3). (B) No difference in subcellular localization of CD45 between WT and Sptlc2^−/−^ cells. Wild-type and Sptlc2^−/−^ RAW264.7 cells transfected with GFP-tagged dectin-1 (green) were incubated with zymosan A particles (blue) on ice, warmed to 37°C for 2 min to initiate phagocytosis, fixed, and then stained with antibodies against CD45 (purple). Boxed areas are magnified at right. Arrowheads mark sites of zymosan A binding. Bar, 5 μm. (C) WT and Sptlc2^−/−^ RAW264.7 cells were stained with APC-conjugated anti-CD45 antibody, fixed, and subjected to flow cytometry analysis. (D) Quantitative analysis of median fluorescence intensity of cells treated as for panel C. (E) Wild-type and Sptlc2^−/−^ RAW264.7 cells were stained with FITC-conjugated anti-dectin-1 antibody, fixed, and subjected to flow cytometry analysis. (F) Quantitative analysis of median fluorescence intensity of cells treated as for panel E. (G) Immunoblot analysis of total cell lysates collected from wild-type and two independent UGCG^−/−^ KO clones of THP-1 cells using antibodies against UGCG and GAPDH. Download FIG S3, TIF file, 0.3 MB. Copyright © 2021 Niekamp et al. 2021 Niekamp et al. This content is distributed under the terms of the Creative Commons Attribution 4.0 International license.

10.1128/mBio.03141-20.6 MOVIE S3 Time-lapse confocal images of a wild-type RAW264.7 cell transfected with dectin-1-GFP (green) and infected with mCherry-expressing M. marinum (red). Images were taken every 15 seconds. Bar, 5 μm. Download Movie S3, AVI file, 0.6 MB. Copyright © 2021 Niekamp et al. 2021 Niekamp et al. This content is distributed under the terms of the Creative Commons Attribution 4.0 International license.

10.1128/mBio.03141-20.7 MOVIE S4 Time-lapse confocal images of a wild-type RAW264.7 cell transfected with dectin-1-GFP (green) and infected with mCherry-expressing M. marinum (red). Images were taken every 15 s. The imaging is similar to that in Movie S3 with a different field. Bar, 5 μm. Download Movie S4, AVI file, 0.9 MB. Copyright © 2021 Niekamp et al. 2021 Niekamp et al. This content is distributed under the terms of the Creative Commons Attribution 4.0 International license.

To address whether a block in sphingolipid biosynthesis affects the overall surface levels of CD45 and dectin-1, wild-type and Sptlc2^−/−^ RAW264.7 cells were stained with allophycocyanin (APC)-conjugated anti-CD45 and fluorescein isothiocyanate (FITC)-conjugated anti-dectin-1 antibodies and subjected to analytical flow cytometry. We found no differences in the overall fluorescent signals for both markers between wild-type and Sptlc2^−/−^ cells (Fig. S3C to F), indicating that the cell surface levels of endogenous dectin-1 and CD45 are unaffected by blocking sphingolipid biosynthesis. From this, we conclude that an intact sphingolipid biosynthetic pathway is required to enable a proper displacement of CD45 from the site of dectin-1 clustering, a step critical for the initiation of phagocytic synapse formation.

### Activation of Rho GTPases Rac1 and Cdc42 is impaired in Sptlc2^−/−^ cells.

**FIG 5:**
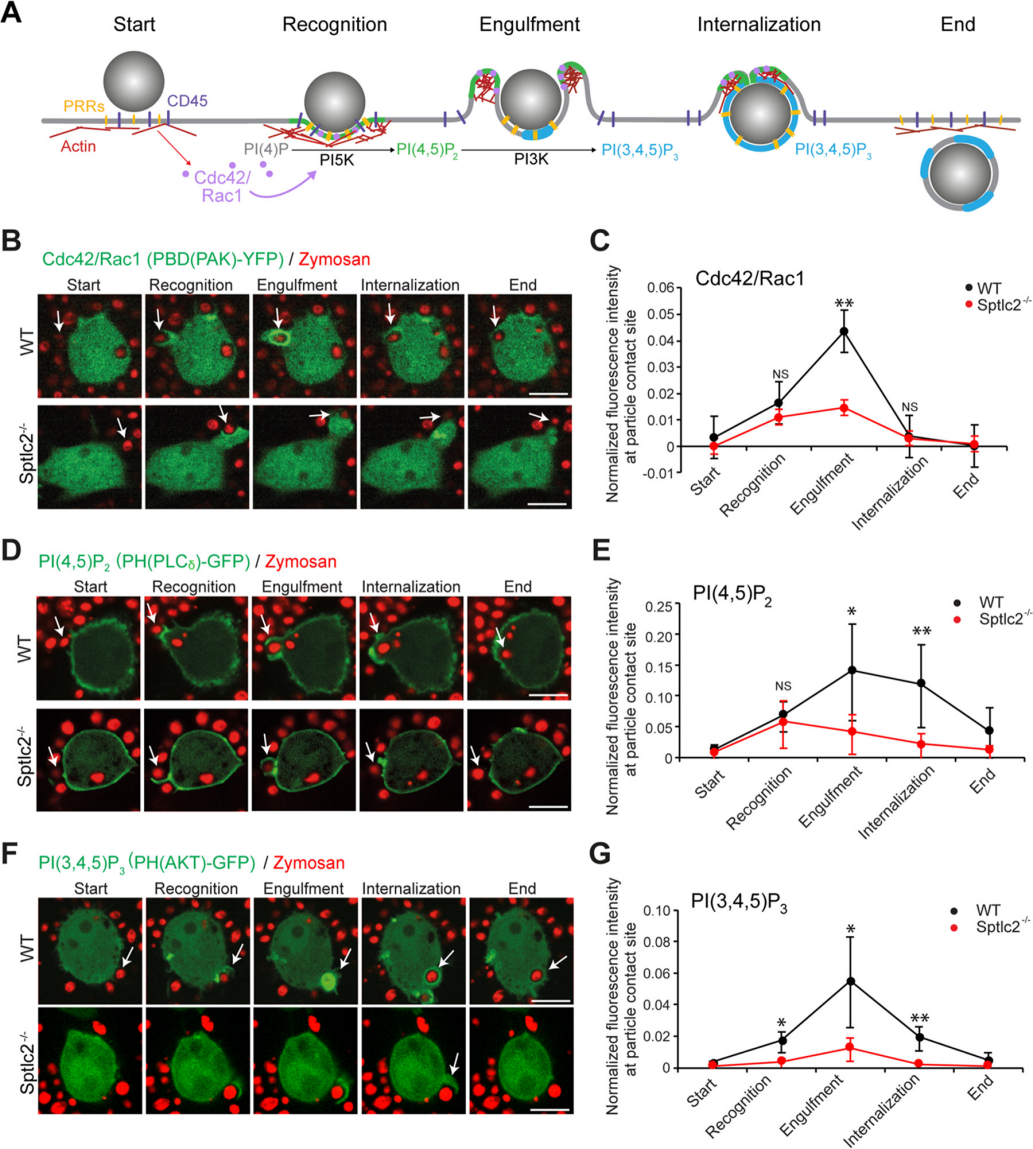
Sptlc2^−/−^ cells display an impaired phosphoinositide turnover at the phagocytic cup. (A) Schematic outline of molecular events underlying phagocytosis. Engagement of pattern recognition receptors (PRRs) with a pathogen initiates receptor clustering at the phagocytic cup, followed by a lateral segregation of the inhibitory CD45 phosphatase. This results in activation of the Rho GTPases Cdc42 and Rac1, which, in turn, stimulate PI5K activity to enable local production of PI(4,5)P_2_ and promote actin assembly. Next, PI3K orchestrates the turnover of PI(4,5)P_2_ to PI(3,4,5)P_3_, leading to actin disassembly at the base of the phagosome, a process required to complete the engulfment and internalization of the pathogen. (B) Time-lapse confocal images of wild-type and Sptlc2^−/−^ RAW264.7 cells transfected with Cdc42 activity biosensor PAK(PBD)-GFP (green) and incubated with zymosan beads (red) at an MOI of 10. (C) Quantification of the PAK(PBD) fluorescence intensity in the course of phagocytic cup formation in cells treated as for panel B. (D) Time-lapse confocal images of wild-type and Sptlc2^−/−^ RAW264.7 cells transfected with the PI(4,5)P_2_ biosensor PH(PLCδ)-GFP (green) and incubated with zymosan beads (red) as for panel B. (E) Quantification of the PH(PLCδ) fluorescence intensity in the course of phagocytic cup formation in cells treated as for panel D. (F) Time-lapse confocal images of wild-type and Sptlc2^−/−^ RAW264.7 cells transfected with the PI(3,4,5)P_3_ biosensor PH(AKT)-GFP (green) and incubated with zymosan beads (red) as for panel B. Bar, 5 μm. (G) Quantification of the PH(AKT) fluorescence intensity in the course of phagocytic cup formation in cells treated as for panel F. Note that only a small fraction of the beads recognized by Sptlc2^−/−^ mutant cells were internalized, in contrast to wild-type cells. Therefore, for Sptlc2^−/−^ mutant cells, the normalized fluorescence intensity was determined at the particle contact site at time points equivalent to those at which particle recognition, engulfment, and internalization took place in wild-type cells. The GFP signal at the particle contact site was quantified and divided by the value for the GFP threshold of the whole cell at the corresponding time point. Average intensity profiles of 5 to 10 cells taken from two independent biological experiments are shown. Data are means ± SD. *, *P* < 0.05; **, *P* < 0.01; ns, not significant (two-tailed unpaired *t* test).

Phagocytosis is strictly regulated through a signaling network that involves the recruitment of pathogen receptors and Rho GTPases to the particle contact site. Activated Rho GTPases promote the conversion of phosphatidylinositol-4-phosphate (PI4P) to phosphatidylinositol-4,5-*bis*-phosphate [PI(4,5)P_2_] by stimulating phosphatidylinositol 5-kinase (PI5K). The local accumulation of PI(4,5)P_2_ is essential to initiate the polymerization of F-actin, a process that drives extension of the leading edges of the nascent phagosome around the particle (38). However, to complete the engulfment and internalization of the particle, F-actin must disassemble at the base of the phagocytic cup. This is achieved through a local conversion of PI(4,5)P_2_ to phosphatidylinositol-3,4,5-*tris*-phosphate [PI(3,4,5)P_3_] by the phosphatidylinositol 3-kinase (PI3K) (Fig. 5A) (5, 38, 39).

To determine whether blocking sphingolipid biosynthesis perturbs phagocytic signaling events downstream of pathogen receptor activation, we first focused on the activation and localization of the Rho GTPases Rac1 and Cdc42 in zymosan A-treated wild-type and Sptlc2^−/−^ RAW264.7 cells. Visualization of activated Rho GTPases was enabled by transfecting cells with a fluorescent biosensor comprising yellow fluorescent protein (YFP) fused to the p21-binding domain of the p21-activated kinase PAK [PBD(PAK)-YFP] (38, 40). At 24 h posttransfection, cells were incubated with zymosan A beads at an MOI of 10 and subjected to live-cell imaging. In wild-type cells, we observed a sharp increase in PBD(PAK)-YFP fluorescence at the particle contact site. Fluorescence levels peaked during particle engulfment, corresponding to the expected pattern of pathogen-induced Rac1/Cdc42 activation (Fig. 5B and C; Movie S5). In Sptlc2^−/−^ cells, we also observed an increase in PBD(PAK)-YFP fluorescence upon particle recognition. However, the PBD(PAK)-YFP fluorescence intensity failed to reach the levels detected in zymosan A-treated wild-type cells and faded out prior to a successful particle internalization, suggesting that Sptlc2^−/−^ cells are impaired in particle-induced activation of Rho GTPases (Fig. 5B and C; Movie S5).

10.1128/mBio.03141-20.8 MOVIE S5 Time-lapse confocal images of a Sptlc2^−/−^ RAW264.7 cell transfected with PAK(PBD)-GFP (green) and incubated with zymosan beads (red). Images were taken every 15 s. Bar, 5 μm. Download Movie S5, AVI file, 0.5 MB. Copyright © 2021 Niekamp et al. 2021 Niekamp et al. This content is distributed under the terms of the Creative Commons Attribution 4.0 International license.

### Phosphoinositide turnover is impaired in Sptlc2^−/−^ cells.

As PI(4,5)P_2_ and PI(3,4,5)P_3_ are key regulators of F-actin dynamics during phagocytosis (Fig. 5A), we next monitored the spatial distribution of these phosphoinositides in zymosan A-treated wild-type and Sptlc2^−/−^ RAW264.7 cells. To this end, cells were first transfected with a GFP fusion of the pleckstrin homology (PH) domain of the phospholipase Cδ [PH(PLCδ)-GFP], which serves as a fluorescent biosensor for PI(4,5)P_2_ (5, 41). In zymosan A-treated wild-type cells, PH(PLCδ)-GFP was readily concentrated at the particle contact site and preferentially accumulated at the leading edges of the nascent phagosome during particle engulfment (Fig. 5D and E; Movie S6). In Sptlc2^−/−^ cells, however, PH(PLCδ)-GFP was readily recruited to the contact site but remained associated with the base of the phagocytic cup, analogous to what we observed with the LifeAct-GFP probe (Fig. 3C). This suggests that PI(4,5)P_2_ synthesis is initiated upon zymosan A binding but that PI(4,5)P_2_ turnover at the base of the phagocytic synapse is impaired in Sptlc2^−/−^ cells (Fig. 5D and E).

10.1128/mBio.03141-20.9 MOVIE S6 Time-lapse confocal images of a Sptlc2^−/−^ RAW264.7 cell transfected with PLCd-GFP (green) and incubated with zymosan beads (red). Images were taken every 15 s. Bar, 5 μm. Download Movie S6, AVI file, 0.2 MB. Copyright © 2021 Niekamp et al. 2021 Niekamp et al. This content is distributed under the terms of the Creative Commons Attribution 4.0 International license.

To check whether a block in sphingolipid biosynthesis affects the turnover of PI(4,5)P_2_ into PI(3,4,5)P_3_ in zymosan-A-treated cells, wild-type and Sptlc2^−/−^ RAW264.7 cells were transfected with a GFP fusion of the PH domain of Akt (PH(AKT)-GFP), which serves as a fluorescent biosensor for PI(3,4,5)P_3_ (5, 41). In wild-type cells, PH(AKT)-GFP was readily recruited to the particle contact site, and its membrane association increased sharply during particle engulfment (Fig. 5F and G; Movie S7). In contrast, Sptlc2^−/−^ cells almost completely failed to mobilize PH(AKT)-GFP at the particle contact site (Fig. 5F and G; Movie S7). Together, these results indicate that blocking sphingolipid biosynthesis disrupts phosphoinositide metabolism at phagocytic synapses in zymosan A-treated macrophages, notably at the level of PI(4,5)P_2_-to-PI(3,4,5)P_3_ conversion, a step known to be critical for the internalization of pathogenic bacteria by professional phagocytes (42).

10.1128/mBio.03141-20.10 MOVIE S7 Time-lapse confocal images of a Sptlc2^−/−^ RAW264.7 cell transfected with PH(AKT)-GFP (green) and incubated with zymosan beads (red). Images were taken every 15 s. Bar, 5 μm. Download Movie S7, AVI file, 0.3 MB. Copyright © 2021 Niekamp et al. 2021 Niekamp et al. This content is distributed under the terms of the Creative Commons Attribution 4.0 International license.

### Production of sphingomyelin, not glycosphingolipids, is critical for efficient *Mtb* uptake.

**FIG 6:**
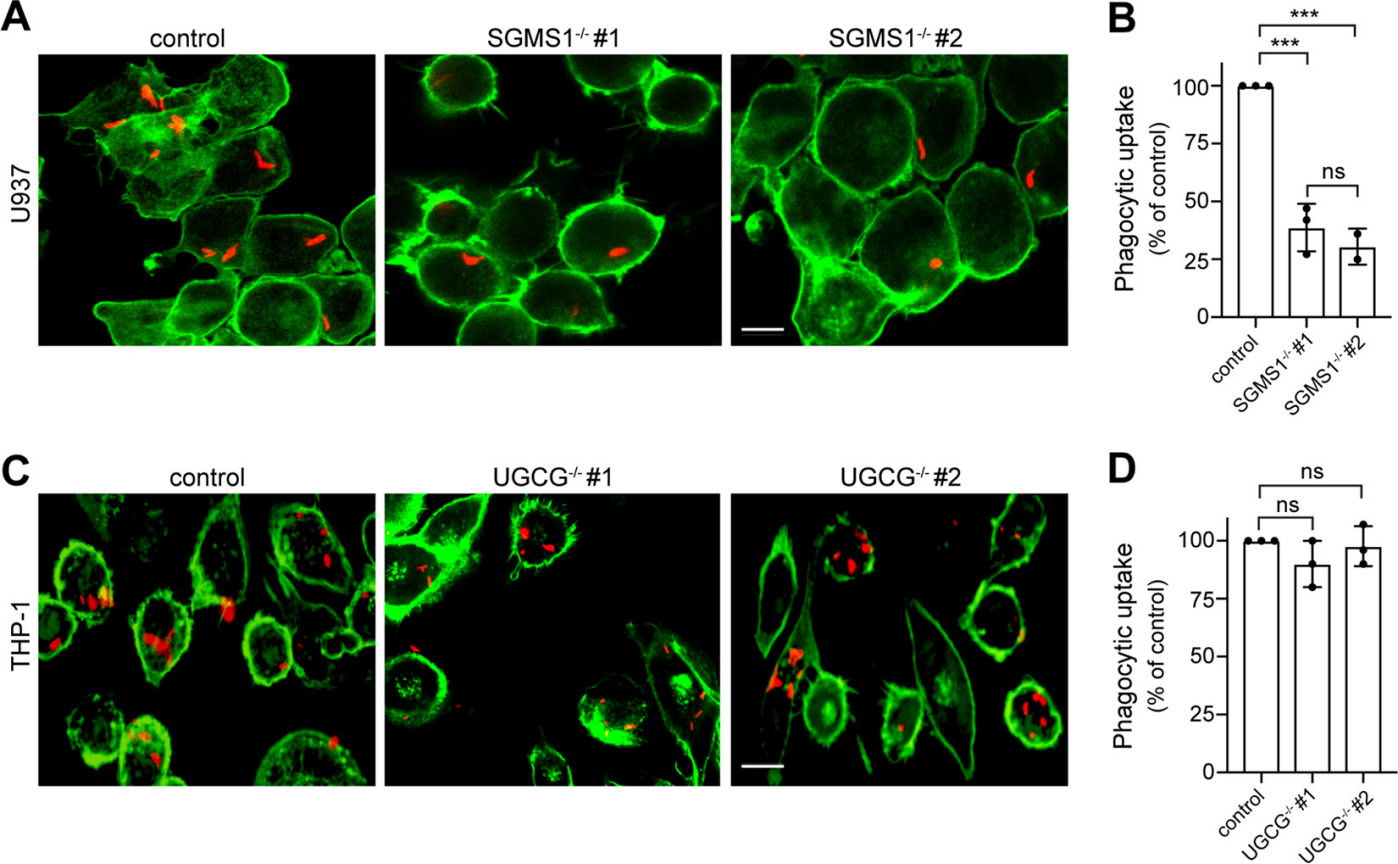
Production of sphingomyelin, not glycosphingolipids, is critical for efficient *Mtb* uptake. (A) Wild-type and two independent SGMS1^−/−^ knockout clones of U937 cells were infected with mCherry-*Mtb* (red) for 2 h, stained with phalloidin Alexa Fluor 488 (green), and then imaged by confocal fluorescence microscopy. (B) Quantification of *Mtb* uptake by cells treated as for panel A. (C) Wild-type and two independent UGCG^−/−^ knockout clones of THP-1 cells were infected with mCherry-expressing *Mtb* (red) for 2 h, stained with phalloidin Alexa Fluor 488 (green), and then imaged by confocal fluorescence microscopy. Bar, 5 μm. (D) Quantification of *Mtb* uptake by cells treated as for panel C. U937 and THP-1 cells were activated with 50 nM PMA for 24 h before *Mtb* infection. Three independent experiments were performed, each in triplicate with at least 1,000 cells quantified per replicate. Data are means ± SD. ***, *P* < 0.001; ns, not significant (two-tailed unpaired *t* test).

Both SM and glycosphingolipids are highly enriched in the exoplasmic leaflet of the plasma membrane of mammalian cells. Therefore, we next addressed whether efficient phagocytic uptake of *Mtb* relies on the production of SM, glycosphingolipids, or both. Bulk production of SM is mediated by the enzyme sphingomyelin synthase 1 (SGMS1) in the *trans*-Golgi, whereas glycosphingolipid production is strictly dependent on the enzyme UDP-glucose ceramide glycosyltransferase (UGCG) in the *cis*-Golgi (24, 43). We used CRISPR (clustered regularly interspaced palindromic repeats)/Cas9 gene editing to ablate UGCG expression in THP-1 cells (Fig. S3G), and the lab of S. V. Winter kindly provided us with SGMS1 knockout U937 cells (44). Strikingly, SGMS1 knockout resulted in a major (∼60%) reduction in the phagocytic uptake of mCherry-expressing *Mtb* by U937 cells (Fig. 6A and B), similar to what we observed in myriocin- or FB1-treated U937 cells (Fig. 2B and C; Fig. S2A to D). In contrast, genetic ablation of UGCG had no impact on *Mtb* uptake by THP-1 cells (Fig. 6C and D), even though phagocytosis of *Mtb* in THP-1 cells can be readily inhibited by myriocin and FB1 (Fig. 2B and C; Fig. S2C and D). Together, these data indicate that efficient uptake of *Mtb* by professional phagocytes is critically dependent on an intact SM biosynthetic pathway, whereas the production of glycosphingolipids is dispensable for this process.

## DISCUSSION

In this study, we report that the invasion of phagocytes by *Mtb* is critically dependent on an intact sphingolipid biosynthetic pathway. Blocking sphingolipid production does not impair clathrin-mediated endocytosis or macropinocytosis of herpes simplex virus, indicating that phagocytes remain susceptible to infection by pathogens that utilize other routes of cellular uptake. Strikingly, sphingolipid-deficient macrophages display normal pathogen-induced clustering of phagocytic receptors but fail to fully exclude the regulatory phosphatase CD45 from the phagocytic synapse. We also show that key steps in the downstream signaling cascade, notably Rho GTPase activation and phosphoinositide turnover, are significantly impaired in the mutant cells, leading to perturbations in actin dynamics and defective phagosome formation. Blocking production of SM, not glycosphingolipids, suffices to disrupt phagocytic uptake of *Mtb*. Collectively, our findings disclose a vital role of SM in an early step of *Mtb* invasion and establish a novel molecular framework for the underlying cellular process.

A major finding of this work is that macrophages defective in sphingolipid biosynthesis exhibit an impaired segregation of CD45 from pathogen-induced clusters of dectin-1 at the phagocytic cup. Dectin-1 belongs to a family of phagocytic cell surface receptors that participate in the internalization of pathogens, including *Mtb* (8, 35, 45) and M. marinum (this study). Upon pathogen engagement, these receptors cluster in the lateral plane of the membrane, bringing their cytosolic domains in close apposition (46). The cytosolic domain of dectin-1 contains a hemi-immunoreceptor tyrosine-based activation motif (hemITAM), which becomes phosphorylated by Src and Syk family kinases upon receptor coalescence (8, 47). Importantly, segregation of the regulatory tyrosine phosphatase CD45 from the site of dectin-1 clustering is a crucial step in the amplification of a downstream phosphorylation cascade (8), which eventually culminates in the recruitment of phosphoinositide-modifying enzymes, including the phosphoinositide 3-kinase (PI3K) (5, 48). Thus, our finding that Sptlc2^−/−^ macrophages fail to generate PI(3,4,5)P_3_ at the phagocytic cup might be due to an impaired phagocyte receptor signaling caused by a defective segregation of CD45. This is consistent with our immunoblot data, which show that sphingolipids are essential for phosphorylation of PI3K during phagocytosis. In particular, we found that blocking the *de novo* sphingolipid biosynthesis using myriocin in THP-1 cells resulted in a 3.5-fold reduction in phosphorylation of PI3K compared to that in control cells (*P* < 0.01; *n *= 3). PI3K activation and formation of PI(3,4,5)P_3_ at the pathogen engagement site are necessary for late stages of pseudopodium progression, the clearance of actin at the base of the phagocytic cup, and phagosome closure (5). Indeed, time-lapse microscopy revealed that these processes are all significantly compromised in Sptlc2^−/−^ macrophages. Collectively, our data suggest that blocking sphingolipid biosynthesis affects the phagocytic uptake of *Mtb* by precluding a proper activation of phagocyte receptors during pathogen engagement.

Another key observation is that production of SM rather than sphingolipid biosynthesis *per se* is critical for efficient phagocytic uptake of *Mtb*. SM is an abundant sphingolipid in the exoplasmic leaflet of the plasma membrane in mammalian cells. Owing to its saturated nature and hydrogen-bonding capacity, SM enables the formation of cholesterol-enriched microdomains (13). In this capacity, SM may play a critical role in the lateral organization and activation of signaling complexes that promote *Mtb* invasion, consistent with prior reports indicating that cholesterol is essential for *Mtb* entry into macrophages (20). However, an alternative scenario is that the phagocytic uptake of *Mtb* relies on the local generation of the SM-derived metabolite ceramide. Previous work revealed that hydrolysis of cell surface SM by acid SMase to liberate ceramide mediates the invasion of Gram-negative pathogens like Neisseria gonorrhoeae and Pseudomonas aeruginosa in mammalian cells (16, 49, 50). Activation of acid SMase at the pathogen contact site appears to trigger local assembly of ceramide-enriched signaling platforms that initiate internalization (17). While SMases and ceramides have also been implicated in mycobacterial infection (51–53), their role in the phagocytic uptake of *Mtb* remains to be established.

We previously reported that blocking sphingolipid biosynthesis in dendritic DC2.4 cells impairs phagocytosis of Candida albicans by preventing the assembly of F-actin at the particle contact site (18). Moreover, the sphingolipid-deficient cells were unable to bind fungal particles and displayed a reduced number of dectin-1 receptors on their surface in comparison to controls. Here, we found that in sphingolipid-deficient RAW264.7 macrophages, which have normal dectin-1 levels, F-actin readily assembles at the particle contact site but fails to undergo subsequent remodeling to complete engulfment of the particle. Thus, sphingolipids in macrophages are required at a step in the phagocytic pathway downstream of particle recognition and receptor clustering. In dendritic cells, this requirement would be masked by a critical role of sphingolipids in maintaining an adequate number of dectin-1 receptors on their surface to enable actin polymerization at the particle recognition site. Why dectin-1 levels in macrophages are not affected by a loss of sphingolipids remains to be established. However, it is well known that the expression profiles of pattern recognition receptors vary considerably among different types of immune cells (54), and that macrophages expose a higher number of dectin-1 receptors on their surface than dendritic cells (55, 56). As these cell types likely also have different lipid compositions, it is conceivable that a block in sphingolipid biosynthesis differentially affects dectin-1 trafficking and/or turnover.

A 2-fold reduction in phagocytic uptake of *Mtb* caused by a block in sphingolipid biosynthesis reported in here appears rather modest. However, it should be noted that both Sptlc2^−/−^ mutant and myriocin-treated phagocytes still contain substantial amounts of SM, i.e., 30 to 40% of the amount found in control cells. As Sptlc2^−/−^ mutant cells rely on serum-supplemented medium for growth (our unpublished data), the residual amount of SM in these cells is likely derived from the serum. Consequently, our data may underestimate the actual functional relevance of SM in the phagocytic uptake of *Mtb*. While the therapeutic value of blocking sphingolipid production in the treatment of TB remains to be established, an ongoing clinical trial investigating the use of statins (a family of cholesterol-depleting drugs) as a synergistic cotherapeutic among a cocktail of frontline antitubercular agents has yielded encouraging results (49).

In sum, we show that *Mtb* relies on an intact SM biosynthetic pathway to efficiently invade professional phagocytes. While the latter process is critical for the clearance of pathogens, *Mtb* harnesses phagocytosis to gain entry into host immune cells and establish long-term survival. By offering fresh insights into the underlying mechanisms, our work provides a basis for the development of new strategies to fight this ancient pathogen.

## MATERIALS AND METHODS

### Reagents.

Myriocin and FB1 were purchased from Cayman Chemical Company. 3-l-[^14^C]serine was purchased from American Radioactivity. Phorbol 12-myristate 13-acetate (PMA) was purchased from BioLegend. Paraformaldehyde was purchased from Fisher Scientific. DyLight phalloidin 488 was purchased from Cell Signaling Technology, and Alexa Fluor 594- and 647-conjugated zymosan A (Saccharomyces cerevisiae) BioParticles were purchased from Thermo Fisher. Mouse monoclonal antibody against GAPDH (ab8245) was purchased from Abcam. Anti-rabbit IgG horseradish peroxidase (HRP)-linked antibody and anti-mouse IgG HRP-linked antibody were purchased from Cell Signaling. APC-conjugated rat monoclonal antibody against CD45 antibody (clone 30-F11) was purchased from BioLegend. Zymosan A BioParticles opsonization reagent was purchased from Invitrogen (no. Z2850). Cy3 AffiniPure donkey anti-rabbit IgG (heavy plus light chain [H+L]) was purchased from JIR (711-165-15). FITC-conjugated rat monoclonal antibody against dectin-1 (clone 2A11) was purchased from AbD Serotec. A Zombie Aqua fixable viability kit (no. 77143) was purchased from BioLegend.

### Plasmids.

The plasmids used for generation of lentivirus consists of the lentiviral vector (pCDH-sgRNA, pCW-Cas9 or pInducer20-LifeAct-mKate), the psPAX2 (packaging) and pMD2.G (envelope) plasmids. pCW-Cas9 plasmid was purchased from Addgene (no. 50661). For generation of pInducer20-LifeAct-mKate, DNA fragments encoding LifeAct-mKate (Addgene no. 54697) were created by PCR and inserted via KpnI and NotI sites into pENTR11 (Invitrogen). Using the Gateway cloning system (Thermo Fisher Scientific), the LifeAct-mKate fragment was transferred onto the lentiviral pInducer20 plasmid (Addgene no. 44012). pMXsIP-Dectin-1-GFP was kindly provided by the lab of Hidde Ploegh (Boston Children’s Hospital, Boston, MA) (8). Active (GTP-bound) Rac1/Cdc42 was detected with PAK(PBD)-YFP, a plasmid encoding the PBD of PAK fused to YFP (40) obtained from Addgene (no. 11407). PI(4,5)P_2_ was detected with PH(PLCδ)-GFP, a plasmid encoding the PH domain of PLCδ fused to GFP. PI(3,4,5)P_3_ levels were monitored by PH(AKT)-GFP, a plasmid encoding the PH domain of AKT fused to GFP (57). These two fusion constructs were kindly provided by the lab of Hidde Ploegh (Boston Children’s Hospital, Boston, MA).

### Cell culture and small-molecule-inhibitor treatment.

Human THP-1 monocytes (ATCC catalog no. TIB-22), human U937 monocytes (ATCC catalog no. CRL-1593.2), and mouse dendritic DC2.4 cells (Millipore catalog no. SCC142) were routinely cultured in RPMI medium supplemented with 10% FBS (Seradigm) and 1% penicillin-streptomycin (Pen/Strep) (Gibco). Mouse RAW264.7 macrophages (ATCC catalog no. TIB-71) were cultured in Dulbecco’s modified Eagle medium (DMEM) supplemented with 10% fetal bovine serum (FBS) and 1% Pen/Strep. All cell lines were cultured at 37°C and 5% CO_2_. Adherent cells (RAW264.7 and DC2.4), were trypsinized using 0.25% trypsin solution (Gibco) and counted using a hemocytometer and trypan blue solution (Gibco) as a counterstain. THP-1 and U937 cells were differentiated to macrophages in RPMI medium supplemented with 10% FBS and 50 ng/ml PMA. After 24 h, cells were washed and treated with 5 μM myriocin or 5 μM FB1 for 3 days in RPMI supplemented with 10% FBS and 1% Pen/Strep. RAW264.7 and DC2.4 cells were treated similarly with myriocin and FB1; however, 24 h before infection, cells were trypsinized, counted, and seeded at an appropriate density in 96-well plates or on coverslips. Biological replicates represent independent treatments with chemical inhibitors and infections on separate days.

### Metabolic labeling and thin-layer chromatography.

A total of 5 × 10^5^ cells were seeded into a 6-well plate. Cells were labeled with 1 μCi/ml of 3-l-[^14^C]-serine for 4 h in Opti-MEM supplemented with the appropriate inhibitors. Cells were then washed twice with phosphate-buffered saline (PBS), and lipid extraction was done following the method of Bligh and Dyer (58).The methanol/chloroform-lipid extracts were dried with nitrogen gas. Dried lipids were redissolved in several drops of chloroform/methanol (1:2, vol/vol) and loaded on a TLC plate. Lipids were separated by developing the TLC plate first in acetone and then in a mixture of chloroform, methanol, and 25% ammonia solution (50:25:6, vol/vol/vol). Radiolabeled lipids were detected on a Storm 825 Phosphor-Imager (GE Healthcare).

### Lipodome analysis.

Liquid chromatography-tandem mass spectrometry (LC-MS/MS) analysis was performed as described previously (59). Lipids were extracted from lysed RAW246.7 cells according to 50 μg of protein by chloroform/methanol extraction. Before the extraction, a standard mix containing sphingosine (d17:1), ceramide (d18:1/17), glycosyl(β) (C_12_ Cer), and sphingomyelin (17:0) was used to spike each sample for normalization and quantification. The dried lipid films were dissolved in a mixture of mobile phase A (60:40, water/acetonitrile, including 10 mM ammonium formate and 0.1% formic acid) and mobile phase B (88:10:2, 2-propanol/acetonitrile/H_2_O, including 2 mM ammonium formate and 0.02% formic acid) with a ratio of 65:35. High-performance liquid chromatography (HPLC) analysis was performed using a C_30_ reverse-phase column (Thermo Acclaim C30; 2.1 by 250 mm; 3 μm; operated at 50°C; Thermo Fisher Scientific) connected to an HP 1100 series HPLC system (Agilent) and a QExactivePLUS Orbitrap mass spectrometer (Thermo Fisher Scientific) equipped with a heated electrospray ionization (HESI) probe. The elution was performed with a gradient of 45 min; during 0 to 3 min, elution started with 40% B and increased to 100% in a linear gradient over 23 min; 100% B was maintained for 3 min. Afterward, solvent B was decreased to 40% and maintained for another 15 min for column re-equilibration. The flow rate was set to 0.1 ml/min. MS spectra of lipids were acquired in full-scan, data-dependent MS2 mode. The maximum injection time for full scans was 100 ms, with a target value of 3,000,000 at a resolution of 70,000 at *m/z* 200 and a mass range of 200 to 2,000 *m/z* in both positive and negative mode. The 10 most intense ions from the survey scan were selected and fragmented with high-energy collisional dissociation (HCD) with a normalized collision energy of 30. Target values for MS/MS were set at 100,000, with a maximum injection time of 50 ms at a resolution of 17,500 at *m/z* 200. To avoid repetitive sequencing, the dynamic exclusion of sequenced lipids was set at 10 s. Peaks were analyzed using the Lipid Search algorithm (MKI, Tokyo, Japan). Peaks were defined through raw files and product ion and precursor ion accurate masses. Candidate molecular species were identified by a database (>1,000,000 entries) search of positive (+H^+^; +NH4^+^) ion adducts. Mass tolerance was set to 5 ppm for the precursor mass. Samples were aligned within a time window, and results were combined in a single report. From the intensities of lipid standards and lipid classes, absolute values for each lipid, in picomoles per milligram of protein, were calculated.

### Generation of lentiviruses.

Lentivirus production was performed as described previously (60). Briefly, lentiviruses were produced by cotransfection of HEK293T cells with lentiviral transfer vectors containing the gene of interest (i.e., pCDH-sgRNA, pCW-Cas9, and pInducer20-LifeAct-mKate) and the packaging plasmids psPAX2 and pMD2.G. Transfection was performed with Lipofectamine 3000 (Thermo Fisher) according to the manufacturer’s instructions. Cells were cultured in DMEM supplemented with 10% FBS, and the growth medium was replaced after 6 h. At 48 h after transfection, lentivirus-containing supernatants were harvested, centrifuged for 5 min at 1,250 rpm, and filtered through a 0.45-μm filter.

### Generation of CRISPR/Cas9-mediated knockout cell lines.

CRISPR/Cas9-mediated genome editing for genes involved in the sphingolipid pathway were performed as described previously (18). Briefly, to generate the Sptlc2^−/−^ knockout, RAW264.7 cells were infected with lentivirus (pCW-Cas9) encoding Cas9 cDNA and were cultured in medium containing 2 μg/ml of puromycin (Sigma-Aldrich). Potential target sequences for CRISPR interference were found with the rules outlined by Mali et al. (61). The following two seed sequences (CRISPR target sequences) preceding PAM motifs found in the open reading frame of Sptlc2 gene were used: Sptlc2 #1, GAACGGCTGCGTCAAGAAC; Sptlc2 #2, AGCAGCACCGCCACCGTCG. The CRISPR target sequence for UGCG was GCTGTGGCTGATGCATTTCA. Potential off-target effects of the seed sequence were evaluated using the NCBI Mus musculus nucleotide BLAST. The CRISPR/Cas9-mediated Sptlc2-knockout RAW264.7 cell line and UGCG-knockout THP-1 cell line were generated as previously described (60). Briefly, CRISPR gBlock was designed to be cloned into the NheI/BamHI site of pCDH-CMV(−) (SBI; CD515B-1) as follows: cacagtcagacagtgactcaGTGTCACAgctagcTTTCCCATGATTCCTTCATATTTGCATATACGATACAAGGCTGTTAGAGAGATAATTAGAATTAATTTGACTGTAAACACAAAGATATTAGTACAAAATACGTGACGTAGAAAGTAATAATTTCTTGGGTAGTTTGCAGTTTTAAAATTATGTTTTAAAATGGACTATCATATGCTTACCGTAACTTGAAAGTATTTCGATTTCTTGGCTTTATATATCTTGTGGAAAGGACGAAACACCGnnnnnnnnnnnnnnnnnnnGTTTTAGAGCTAGAAATAGCAAGTTAAAATAAGGCTAGTCCGTTATCAACTTGAAAAAGTGGCACCGAGTCGGTGCTTTTTTTggatccTGTGCACAgtcagtcacagtcagtctac (n, CRISPR target sequence; lowercase indicates extra nucleotides added to optimize synthesis or restriction enzyme sites). The gBlock was then digested using the restriction enzymes NheI and BamHI and ligated into the pCDH-CMV(−) vector, which had been linearized by digestion with the same restriction enzyme. The Cas9-inducible cells were infected with lentivirus carrying pCDH-CMV(−)-sgRNA and were cultured in medium containing 250 μg/ml of hygromycin B (Life Technology). To induce expression of Cas9, cells were treated with 1 μg/ml of doxycycline (Clontech) for 3 to 5 days. Clonal selection was performed by single-cell dilution on 96-well plates. The individual colonies were collected, and the expression of Sptlc2 was examined by immunoblotting using an anti-Sptlc2 antibody. U937 SGSM1^−/−^ cells were kindly provided by Arturo Zychlinsky (Max Planck Institute of Biochemistry, Berlin, Germany), and the CRISPR/Cas9-mediated knockout cell lines were established as described by Winter et al. (44).

### Culturing of *Mtb* and M. marinum.

Culturing and infection of *Mtb* were conducted in a biosafety level 3 laboratory following general safety guidelines. *Mtb* strain H37R, constitutively expressing mCherry, was obtained from the Fortune lab (Ragon Institute, Cambridge, MA). *Mtb* was cultured with shaking at 150 rpm at 37°C in 7H9 broth medium supplemented with 50 μg/ml hygromycin B. Culturing and infection of M. marinum were conducted in a biosafety level 2 laboratory following general safety guidelines. M. marinum constitutively expressing mCherry was cultured with shaking at 150 rpm in 7H9 medium supplemented with 10% oleic acid-albumin-dextrose-catalase (OADC), 0.05% Tween 80, 0.2% glycerol, and 50 μg/ml hygromycin B at 32°C. To minimize clumping of the bacteria, Erlenmeyer flasks containing 5 mm glass beads were used. For infections, cultures with an optical density at 600 nm (OD_600_) of 0.8 to 1.0 were used.

### *Mtb* and M. marinum infection.

For infection of THP-1 and U937 cells, the cells were seeded in SensoPlate 96-well glass-bottom plates (Greiner Bio-One) at a density of 1.5 × 10^4^ cells per well in RPMI supplemented with 10% FBS, 1% Pen/Strep and 50 ng/ml PMA at 37°C. One day after differentiation, cells were treated for 3 days with 5 μM myriocin, 5 μM FB1, or the vehicle. For infection of RAW264.7 and DC2.4 cells, the cells were cultured in RPMI supplemented with 10% FBS and 1% Pen/Strep and treated for 3 days before infection with 5 μM myriocin, 5 μM FB1, or the vehicle. At 24 h before infection, RAW264.7 and DC2.4 cells were used to seed SensoPlate 96-well glass-bottom plates (Greiner Bio-One) at a density of 2 × 10^4^ cells per well. Before infection, the mycobacteria were first passaged through a 5-μm filter (SLSV025LS; Merck) and then passaged 10 times through a 25-gauge needle to avoid clumping. All cell lines and conditions were then infected with a multiplicity of infection (MOI) of 10 in RPMI supplemented with 10% FBS for 2 h at 37°C. Myr, FB1, and vehicle were kept during infection. Cells were washed with PBS and then fixed with 4% paraformaldehyde (PFA) overnight at 4°C.

### Zymosan A uptake assay.

For infection of RAW264.7 cells, 2.0 × 10^4^ cells per well were seeded in SensoPlate 96-well glass-bottom plates (Greiner Bio-One). After 24 h, the cells were incubated with zymosan A beads at an MOI of 10 in RPMI supplemented with 10% FBS for 1 h at 37°C. Cells were washed with PBS and then fixed with 4% PFA overnight at 4°C.

### Phagocytosis assay.

The phagocytosis assay was performed as previously described (62). Briefly, fixed cells were washed twice with PBS and incubated for 15 min in permeabilization buffer (0.1% Triton X-100 and 1% bovine serum albumin [BSA]). Cells were stained with phalloidin-Alexa Fluor 488 (Thermo Fisher) at a final concentration of 33 nM in permeabilization buffer for 60 min and washed once with PBS. After the phalloidin staining, cells were stained with DAPI for 10 min and washed 3 times with PBS. Cells were then imaged using a Keyence BZ-X700 microscope with a PlanFluor 20× objective. The parameters for imaging were kept the same for each sample. For image analysis, Keyence BZ-X Analyzer software was used. In brief, the outline of the cells was determined via the Alexa Fluor 488 signal (phalloidin staining), and the number and area of mCherry (*Mtb* and M. marinum) or Alexa Fluor 594 (zymosan A) signals in the Alexa Fluor 488 signal were detected by the program. In a second round, the number of cells was determined by counting the nuclei via DAPI staining. The number of bacteria or particles (mCherry or A594 signal) inside the cell was divided by the number of cells (DAPI signal) to identify the phagocytosis efficiency.

### Herpes simplex virus 1 infection.

HSV-1–mCherry reporter virus was kindly provided by the lab of David Johnson (Oregon Health and Science University, Portland, OR). Twenty-four hours prior to infection, 1 × 10^4^ wild-type and Sptlc2 knockout RAW264.7 cells were seeded on glass coverslips in a 12-well plate. Cells were equally infected at an MOI of ∼1 and incubated for 12 h at 37°C and 5% CO_2_. Coverslips were fixed for 20 min in 4% paraformaldehyde, stained with DAPI, and then imaged on a Keyence BZ-X700 microscope. Infection rates were calculated by dividing the mCherry-expressing cells by the total number of cells (DAPI signal).

### Binding assay zymosan A particles.

RAW264.7 cells were seeded in SensoPlate 96-well glass-bottom plates at 1.5 × 10^4^ cells/well 24 h prior to the experiment. Before the experiment, unlabeled zymosan A particles were incubated with zymosan A opsonizing reagent (rabbit polyclonal IgG; Invitrogen) for 1 h at 37°C. The particles were washed 3 times with PBS and resuspended in RPMI supplemented with 10% FBS. Cell growth medium was replaced with medium containing opsonized zymosan A particles at an MOI of 10. The 96-well plates were centrifuged at 1,000 rpm for 30 s. Cells were incubated at 37°C for 10 min. Next, cells were placed on ice, washed twice with chilled PBS, and stained for 15 min at 4°C for external zymosan A particles with anti-rabbit-Cy3 (JIR). Afterwards, cells were fixed with 4% PFA for 15 min and stained with phalloidin Alexa Fluor 488 for 1 h. The ratio of internalized (blue) beads to attached beads (red beads with clear phalloidin Alexa Fluor 488 signal) was quantified manually.

### Real time imaging of phagocytosis.

For transient transfection, RAW264.7 cells were seeded in SensoPlate 96-well glass-bottom plates at 1.5 × 10^4^ cells/well 24 h prior to live imaging. Transfection was conducted with Lipofectamine 3000 according to manufacturer’s instructions 16 h prior to imaging. Alternatively, RAW 264.7 cells containing a doxycycline-inducible LifeAct-mKate construct were seeded in SensoPlate 96-well glass-bottom plates at 1.5 × 10^4^ cells/well 24 h prior to transfection, and the medium was supplemented with 1 μg/ml doxycycline. For imaging, the growth medium was aspirated and replaced with RPMI without phenol red, supplemented with 30 mM HEPES and containing mCherry-expressing M. marinum at an MOI of 25 or zymosan A BioParticles with Alexa Fluor 595 at an MOI of 10. The 96-well plates were centrifuged at 1,000 rpm for 30 s. The cells were imaged with an SDC microscope (Nikon), and 3 or 4 different spots per well were imaged at the same time for 60 min with capture intervals of 15 s. The parameters for imaging were kept the same for each sample.

### Live imaging of dectin-1–GFP.

RAW264.7 cells were seeded in SensoPlate 96-well glass-bottom plates at 1.5 × 10^4^ cells/well 24 h prior to live imaging, and transfection was conducted with Lipofectamine 3000 according to the manufacturer’s instructions 16 h prior to imaging. For imaging, the growth medium was aspirated and replaced with RPMI without phenol red, supplemented with 30 mM HEPES, and containing mCherry-expressing M. marinum at an MOI of 25. The 96-well plates were centrifuged at 1,000 rpm for 30 s. The cells were imaged with an SD microscope (Zeiss), and 3 or 4 different spots per well were imaged at the same time for 60 min with capture intervals of 15 s. The parameters for imaging were kept the same for each sample.

### Live image analysis (quantification).

Of live cell images, individual images were selected for further analysis if they captured a cell bound to M. marinum or a zymosan A particle while maintaining a healthy morphology throughout the imaging time course. Imaging analysis was performed using FIJI software (56). The parameters for image processing were kept constant when different data sets were compared. For Fig. 3, all cells were included in the analysis that showed a clear recognition of the bacteria or particles, which was defined by an increase in F-actin signal around the bacterium or particle, and the number of successful versus failed internalizations was quantified. For Fig. 5, all cells were included in the analysis that showed a clear recognition of the zymosan A particles, which was defined by an increase in the corresponding reporter signal at the particle contact site. For quantification of Fig. 5, the area of the particle contact site was selected, cropped, and opened in a new window. The threshold for the particle contact site was set 1.5 times higher than the whole-cell threshold to exclusively detect an increase in fluorescence signal. The fluorescence intensity was measured at different time points by the analyze-measure function of FIJI. The values for the GFP threshold of the particle contact site area were divided by the values for the GFP threshold of the whole cell at the corresponding time points.

### Dectin-1 and CD45 displacement assay.

Raw 264.7 WT and Sptlc2^−/−^ macrophages were seeded on glass coverslips in 24-well plates at a density of 50,000 cells per well and grown overnight. The next day, cells were transfected with dectin-1–GFP using Lipofectamine 3000 (Thermo Fisher) according to the manufacturer’s instructions. At 24 h posttransfection, cells were washed three times with ice-cold PBS, chilled on ice for 5 min, and inoculated with a chilled suspension of zymosan A beads (250 μg/ml in serum-free DMEM, prepared with repeated vortexing). After brief centrifugation (3 min at 250 × *g* and 4°C), plates were incubated at 37°C for 3 min, washed three times with ice-cold PBS, and fixed for 15 min with room-temperature 4% PFA. Cells were permeabilized for 30 min with 0.2% saponin in PBS, incubated for 1 h in blocking buffer (5% normal goat serum, 0.01% saponin in PBS), and stained for 1 h with anti-CD45–APC (BioLegend) diluted 1:100 in blocking buffer. Coverslips were mounted on glass slides with ProLong Glass antifade mountant (Thermo Fisher). Superresolution microscopy was performed on a Zeiss LSM 880 confocal microscope with the Airyscan module using a 63×, 1.4 numerical aperture (NA) oil immersion objective. Airyscan processing was performed with ZEN Black (Zeiss) using the software’s preset parameters. Intensity profiles of dectin-1 and CD45 were created using the 2.5D visualization module. Measurement of CD45 exclusion from phagocytic cups was performed with Imaris 9.5 (Bitplane). All Airyscan images were converted to the .ims file format and subjected to background subtraction using the software’s default settings. The Surfaces module was then used to build three-dimensional (3D) models of phagocytic cups using absolute intensity of dectin-1 signal and a volume cutoff of 1 μm^3^, followed by masking so that all signals outside the cups were set to zero. All images were fed through this Surfaces pipeline, and statistics files were downloaded in the Microsoft Excel format. Percent CD45 exclusion was calculated for each cup as [1 − (number of CD45 voxels/sum of CD45 and dectin-1 voxels)] × 100.

### Immunoblot analysis.

About 1 × 10^6^ THP-1 cells were seeded in a 6-well plate and treated for 3 days with 5 μM myriocin or left untreated. Cells were then infected with *Mtb* (H37Rv) at an MOI of 10. At 10 min postinfection, cells were washed with PBS, lysed, separated by SDS-PAGE, and transferred to a membrane. Levels of PI3K phosphorylation were detected by immunoblot analysis using an anti-phospho-PI3K antibody (p85; clone 19H8; Cell Signaling Technology). Immunostaining with an anti-GAPDH antibody (clone D16H11; Cell Signaling Technology) served as a loading control. Results were from three independent biological replicates and normalized with GAPDH signal. Quantification of the bands was performed using FIJI, and a Student's *t* test was performed to show significance.

### Flow cytometry.

RAW264.7 wild-type and Sptlc2^−/−^ cells were rinsed with PBS and lifted from the flask using treatment with trypsin. Fc receptor was blocked with CD16/32 antibody (1:100), and cells were stained with Zombie Aqua live/dead stain (1:200). Cells were then stained with either CD45-APC (1:200) or dectin-1–FITC (1:50) for 30 min. Finally, cells were fixed for 20 min in 4% paraformaldehyde and analyzed using an LSR-II flow cytometer. Cells independently stained with Zombie Aqua, CD45-APC, and dectin-1–FITC were used for compensation. Analysis was performed using FlowJo software.

### Transferrin uptake analysis.

Undifferentiated THP-1 cells were counted and plated in a SensoPlate 96-well glass-bottom plates (Greiner Bio-One) at a density of 2.5 × 10^4^ in RPMI supplemented with 10% FCS, 1% PS, and 50 ng/ml PMA. Cells were allowed to differentiate at 37°C and 5% CO_2_ for 3 days before being treated with 5 μM myriocin for another 3 days (or left untreated). These cells were then starved for 30 min in serum-free RPMI and treated with 100 nM Alexa Fluor 555-conjugated human transferrin (T35352; Thermo Fisher Scientific) resuspended in serum-supplemented RPMI. After a 30-min uptake, cells were washed once with PBS at pH 7.0 and once with PBS acidified to pH 5.0 (to release surface-bound transferrin) and fixed for 1 h at 4°C using 4% paraformaldehyde. Cells were stained with both DyLight 488-conjugated phalloidin (12935S; Cell Signaling Technology) and DAPI according to the manufacturer’s specifications. Uptake comparison between untreated and myriocin treated cells was quantified using high-content imaging collected on a Keyence BZ-X700 microscope using a Super Fluor 40× oil immersion objective. Using Keyence BZ-X Analyzer software, cell interior was demarcated using the phalloidin signal, nuclear area was assessed using the 405 signal, and transferrin internalization was assessed using the 555 signal. Data are reported as the integrated 555 signal per cell, normalized by the 405-positive nuclear area, with three independent biological experiments and four technical replicates each. A competition assay was performed in the presence of additional nonfluorescent human transferrin (T8158-100MG; Sigma-Aldrich) and quantified with a CLARIOstar Plus fluorimeter (BMG Labtech). Fluorescence emission was collected using a spiral detection pattern averaging across 200 reads per well. Resultant data are reported as the raw fluorescence intensities, in arbitrary fluorescent units.
