# Supplementary figures and images for: Sphingomyelin Biosynthesis Is Essential for Phagocytic Signaling during Mycobacterium tuberculosis Host Cell Entry
Source: mBio. 2021 Jan 26;12(1):e03141-20. doi: 10.1128/mBio.03141-20 (PMC7858061; doi:10.1128/mBio.03141-20)

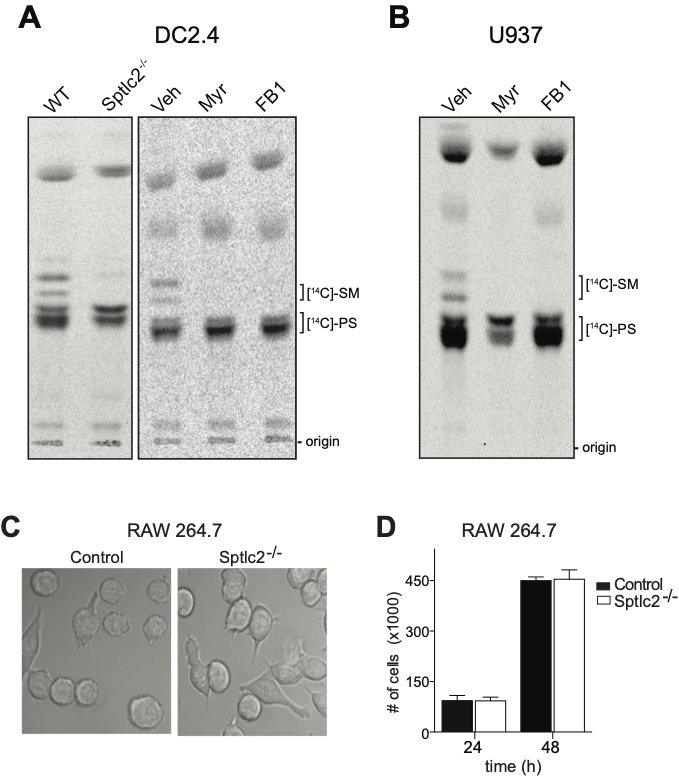

Supplement: FIG S1 [file mBio.03141-20-sf001.tif]
